# Where does it hurt? Small area estimates and inequality in the prevalence of chronic pain

**DOI:** 10.1002/ejp.2148

**Published:** 2023-06-21

**Authors:** Marty Lynch, George Peat, Kelvin Jordan, Dahai Yu, Ross Wilkie

**Affiliations:** ^1^ School of Medicine Keele University Keele UK; ^2^ The Centre for Applied Health & Social Care Research Sheffield Hallam University Sheffield UK

## Abstract

**Background:**

Chronic pain affects up to half of UK adults, impacting quality of life and demand on local health services. Whilst local health planning is currently based on subnational prevalence estimates, associations between pain and sociodemographic characteristics suggest that inequalities in the prevalence of chronic and high‐impact chronic pain between neighbourhoods within local authorities are likely. We aimed to derive lower super output area (LSOA) estimates of the prevalence of chronic and high‐impact chronic pain.

**Methods:**

Presence of self‐reported chronic and high‐impact chronic pain were measured in adults aged 35+ in North Staffordshire and modelled using multilevel regression as a function of demographic and geographic predictors. Multilevel model predictions were post‐stratified using the North Staffordshire age‐sex population structure and LSOA demographic characteristics to estimate the prevalence of chronic and high‐impact chronic pain in 298 LSOAs, corrected for ethnic diversity underrepresented in the data. Confidence intervals were generated for high‐impact chronic pain using bootstrapping.

**Results:**

Data were analysed from 4162 survey respondents (2358 women, 1804 men). The estimated prevalence of chronic and high‐impact chronic pain in North Staffordshire LSOAs ranged from 18.6% to 50.1% and 6.18 [1.71, 16.0]% to 33.09 [13.3, 44.7]%, respectively.

**Conclusions:**

Prevalence of chronic and high‐impact chronic pain in adults aged 35+ varies substantially between neighbourhoods within local authorities. Further insight into small‐area level variation will help target resources to improve the management and prevention of chronic and high‐impact chronic pain to reduce the impact on individuals, communities, workplaces, services and the economy.

**Significance:**

Post‐stratified multilevel model predictions can produce small‐area estimates of pain prevalence and impact. The evidence of substantial variation indicates a need to collect local‐level data on pain and its impact to understand health needs and to guide interventions.

## 1 INTRODUCTION

Chronic pain affects one‐third to one‐half of the adult population of the United Kingdom (Fayaz et al., 2016), and has a substantial impact on the health, well‐being, and quality of life of populations and individuals (Donaldson, 2009). In common with many other long‐term health conditions, there are variations in chronic pain prevalence by age, sex, ethnicity, and socioeconomic status (Mills et al., 2019). The distribution of these and other determinants is expected to contribute to geographical variation, yet there is ‘a near absence of research on the geographic distribution of pain at subnational levels’ (Zajacova et al., 2021).

In England, public health profiles used to inform local health planning currently contain subnational prevalence estimates of musculoskeletal conditions—the largest cause of pain in the adult population (OHID, 2022). Estimates of the most common painful conditions are derived from national surveys, general practice and emergency hospital admissions data and provide evidence of regional variation (Adomaviciute et al., 2018; Todd et al., 2018). The lowest level of granularity is at local authority level, which has been found to mask significant variation within their large and diverse populations (median population of 140,000; Asaria et al., 2016; Sheringham et al., 2017; e.g. neighbourhood‐level estimates for diabetes (Noble et al., 2012), cardiovascular disease (Asthana & Gibson, 2021), and other chronic diseases show two‐ to three‐fold variations in prevalence between neighbourhoods within the same local authority). Inequalities may widen over time within some local authorities whilst simultaneously narrowing in others (Sheringham et al., 2017). More granular detail is needed for local health planners and place‐based partnerships with a duty or concern to reduce health inequalities and who are seeking to better target advice, support, interventions, and services (Charles et al., 2021; Donaldson, 2009; Health and Care Bill, 2021). This includes priority actions highlighted in the NHS Long‐Term Plan and other policy documents such as direct access to First Contact Practitioners in primary care, expansion of physiotherapy provision within Primary Care Networks, access to vocational advice and rehabilitation, and access to digital and face‐to‐face support programmes for pain management.

Multilevel modelling of survey data with poststratification is a well‐established approach outside of health research for modelling characteristics that may vary geographically, without data being necessarily available for every geographic area under study (Kastellec et al., 2019). There may be advantages in the application of this technique when using survey data to generate small area prevalence estimates for health conditions that may not be recorded consistently in primary care or may be present in the local population without seeking of health care (Zhang et al., 2014).

In the current study, we used multilevel regression and post‐stratification applied to information collected from a population survey of adults in three local authorities in England, to derive small‐area estimates (lower super output area [LSOA]) of the prevalence of chronic pain. Specifically, we hypothesised that: (i) within local authorities there are inequalities between neighbourhoods in the prevalence of chronic pain and these are of a similar magnitude to those reported for other non‐communicable diseases and strongly related to age and deprivation; (ii) inequalities are more pronounced for severe and disabling chronic pain (i.e. high‐impact chronic pain). An additional aim was to demonstrate the methods and visualization of findings from such an approach.

## 2 METHODS

### 2.1 Study population and setting

Our target population were adults aged 35 years and over living in three adjacent local authorities—Stoke‐on‐Trent, Newcastle‐under‐Lyme, and Staffordshire Moorlands—located in the county of Staffordshire, in the North Midlands region of England. They cover a combined area of 880 km^2^ and a total population of 480,000 (57% aged over 35 years) with a wide spectrum of population characteristics and living conditions. 40% of North Staffordshire is classed as rural or hub town living, however, 99% of Stoke‐on‐Trent is urban with 30% of its neighbourhoods in the most deprived decile in England. By contrast, 10 of 298 neighbourhoods, mostly in North Staffordshire, are in the most affluent decile; 91% identify as White, with Asian/Asian British as the next most common ethnic group comprising 9% of the population of Stoke‐on‐Trent. The planning and commissioning of healthcare services for the local population is led by two NHS Clinical Commissioning Groups (CCGs) with 71 general practices organized into 13 Primary Care Networks.

For our cross‐sectional survey (June 2017), we used the patient registers of 11 general practices (covering both CCGs) as our sample frame (Jordan et al., 2004). GP registration is needed to access non‐emergency healthcare in England making registers an efficient population sample frame. For our population sample, we selected a random 18%–24% sample of registered patients aged 35 and over at the time of the survey, irrespective of consultation.

### 2.2 Data collection

Eight thousand, four hundred and sixty one eligible participants were mailed a questionnaire enclosed with an invitation letter from their general practice, an information sheet, and prepaid return envelope. A repeat pack was sent to non‐respondents after 2 weeks, offering the option of online questionnaire completion. After a further 2 weeks, non‐respondents were mailed a shortened questionnaire, restricted to selected outcome measures and descriptive fields. Ethical approval was obtained from the North West—Greater Manchester East Research Ethics Committee (Reference: 15/NW/0735).

#### 2.2.1 Outcomes

We used the following binary outcome indicators, consistent with definitions developed and used in the US National Pain Survey (Dahlhamer et al., 2018; Von Korff et al., 2016) and comparable with definitions used in the most recent Health Survey for England (PHE, 2017):

1. Chronic pain: defined as pain on most days or more in the past 6 months, to indicate having pain for more than 3 months. *In the past 6 months, how often did you have pain? (Never, Some days, Most days, Every day)* (Von Korff et al., 2016).
2. High‐impact chronic pain: defined as pain on most days or more in the past 6 months and which limited activities on most days or more over the same period. *Over the past 6 months, how often did pain limit your life or work activities? (Never, Some days, Most days, Every day)* (Von Korff et al., 2016).

#### 2.2.2 Population subgroup identifiers

Age and sex were categorized into the interaction between sex (male/female) with age group (35–44, 45–54, 55–64, 65–74, 75–84, and 85+ years). The reference age × sex category was 35‐ to 44‐year‐old men. Area‐level deprivation was based on residential postcode and classified into 10 categories based on the national ranking of index of multiple deprivation (IMD) 2015 (Ministry of Housing, Communities, & Local Government, 2015). Most affluent (IMD 10) was taken as the reference category. Each LSOA was also classified as urban or rural based on the 2011 Rural–Urban Classification.

Depression was captured by the Hospital Anxiety and Depression Scale (HADS) depression subscale (range 0–21, higher scores indicating greater anxiety/depression). Body mass index (BMI) was calculated as kg/m^2^ from self‐reported body weight and height.

### 2.3 Statistical analysis

Multilevel modelling with poststratification was used to estimate the prevalence of chronic pain and high‐impact chronic pain for each LSOA in North Staffordshire. Presence of chronic pain and high‐impact chronic pain were modelled using multilevel regression as a function of demographic and geographic predictors (Kastellec et al., 2019; Zhang et al., 2014): age × sex (level 1), area‐level deprivation (level 2), rurality (level 2), and variance between LSOA of residence (level 2 varying effect).

$$Y_{\mathrm{ij}}=\beta_{0j}+\beta_{1\mathrm{ij}}+\beta_{2j}+\mu_{0j},$$

Y, whether an individual has chronic pain/high‐impact chronic pain. $\beta_{0j}$, intercept. $\beta_{1\mathrm{ij}}$, covariates measured at the individual level: age × sex. $\beta_{2j}$, covariates measured at LSOA‐level: IMD decile, rurality. $\mu_{0j}$, between‐LSOA variance.

Model coefficients (including LSOA‐estimated varying effects where LSOAs were represented by respondents) were applied to population data to estimate the prevalence of chronic and high‐impact chronic pain for each LSOA.

Analyses were performed on the complete case sample of respondents with data for all individual‐level variables and outcome (age, sex, high‐impact chronic pain, chronic pain). Individual‐level data on self‐reported ethnicity could not be included in models due to insufficient numbers of respondents known to be of Black, Asian, or another minority ethnic background. Instead, correction factors for ethnicity were applied to post‐stratified prevalence estimates to adjust for diversity in the population not reflected in the study sample. Modelled aggregated ethnicity proportions for residents in each of 298 LSOAs across North Staffordshire and Stoke‐on‐Trent were obtained under a User Agreement from the Consumer Data Research Centre Data Service (Approved Project Reference: 749‐01; van Dijk et al., 2020). Available literature and data sources from the UK suggested a higher prevalence of chronic pain and more severe and widespread pain in people from Black, Asian and other ethnic minority groups with possible exception of people identifying as Chinese ethnicity (Allison et al., 2002; Macfarlane et al., 2015; Nicholl et al., 2015; PHE, 2017; University of Essex, 2022). Multiplicative correction factors (compared to prevalence in White populations) used for chronic pain and high‐impact chronic pain, respectively, were: Asian excluding Chinese (1.1, 1.2), Black (1.2, 1.5), Chinese (0.9, 0.8), Mixed/multiple (1.0, 1.1).

To understand the degree of uncertainty around LSOA‐specific estimates from our approach, confidence intervals were generated (using the percentile method) for post‐stratified prevalence estimates of high‐impact chronic pain (the rarer outcome) in each LSOA in North Staffordshire through bootstrapping with 5000 replicates.

Multilevel regression was carried out in MLWin (R package ‘R2MLWin’) using the Monte Carlo Markov Chain method with 50,000 burn‐in iterations and 500,000 stored iterations. Poststratification was carried out in R version 4.1.0. Bootstrapping was performed in parallel in R using functionality from the R package ‘boot’ applied to R2MLWin (Canty & Ripley, 2017; Zhang et al., 2016).

#### 2.3.1 Sensitivity analyses

We conducted two sensitivity analyses. First, we re‐ran the models without the ethnicity correction factors to explore their impact on overall and LSOA‐specific prevalence estimates of chronic and high‐impact chronic pain. Second, we explored the potential to improve the model fit by adding selected individual‐level measures (depression and BMI) collected from the survey and known to be associated with chronic pain and disability. Models with depression, BMI, and both depression and BMI as additional predictors were estimated using a complete case sample excluding respondents with missing depression and/or BMI data. Respondents with four or more missing HADS depression items were considered to have missing data. Respondents with up to three missing items had responses scored from completed items and then prorated to the same scale as those who completed all seven items. Depression and BMI were included in the multilevel models as continuous variables centred around the mean.

### 2.4 Public involvement and engagement

Prior to obtaining ethical approval, we pre‐tested the survey instrument, study documentation, and online platform with members of our Research Users Group (RUG). Questionnaire content and presentation was agreed with the RUG with the aim of optimizing response and with consideration of responder burden (average time to completion 15 min). A separate Patient Advisory Group (*n* = 7) convened to discuss the findings and their interpretation as well as dissemination to the public and relevant stakeholders.

## 3 RESULTS

### 3.1 Sample size

Of the 4389 individuals who completed the survey (adjusted response rate 47.8%), 4162 (2358 women, 1804 men, Table 1) had complete age, sex, chronic and high‐impact chronic pain data. Missingness patterns are shown in Supporting Information (Tables S1 and S2). Descriptive statistics of respondents by sex, age group, rurality, IMD decile, and prevalence of high‐impact chronic pain are shown in Table 1 and Table S3. Figure S1 shows the number of respondents per LSOA in North Staffordshire.

**TABLE 1:** Respondents and number (%) reporting chronic pain and high‐impact chronic pain by sex and age group.

	Respondents	Number reporting chronic pain (%)	Number reporting high‐impact chronic pain (%)
Women			
35–44	198	41 (21)	14 (7)
45–54	451	146 (32)	50 (11)
55–64	558	195 (35)	83 (15)
65–74	693	261 (38)	107 (15)
75–84	368	167 (45)	92 (25)
85+	90	49 (54)	26 (29)
Men			
35–44	103	24 (23)	6 (6)
45–54	313	88 (28)	26 (8)
55–64	437	137 (31)	46 (11)
65–74	555	200 (36)	90 (16)
75–84	325	134 (41)	70 (22)
85+	71	22 (31)	13 (18)

### 3.2 Model coefficients

Model coefficients reflected the anticipated direction of association between chronic pain or high‐impact chronic pain and increasing age, female sex, and more deprived neighbourhood (IMD decile; Table S4). Prevalence estimated from our models was somewhat higher in urban than in rural areas (Tables S5 and S6).

### 3.3 Estimated prevalence of chronic pain

**FIGURE 1:**
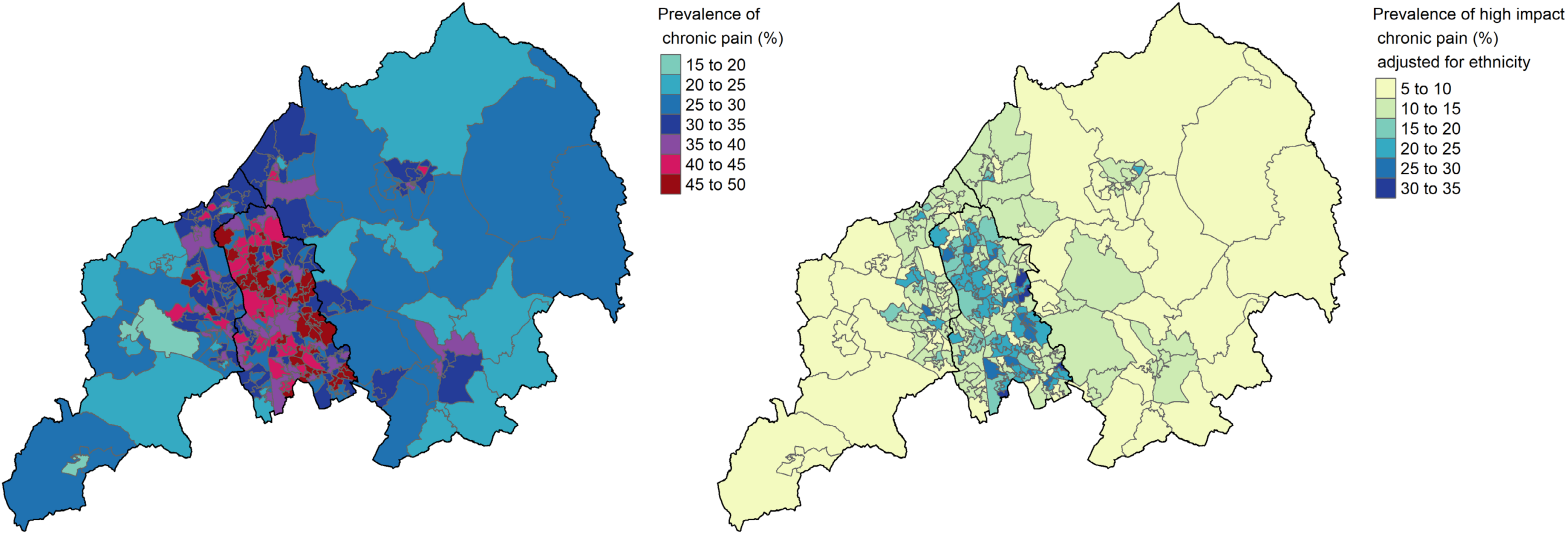
Map of the estimated prevalence of chronic pain (left) and high‐impact chronic pain (right) in Lower Layer Super Output Areas in North Staffordshire and Stoke‐on‐Trent.

The estimated prevalence of chronic pain in North Staffordshire & Stoke‐on‐Trent was 34.5% and ranged from 18.6% to 50.1% (median 33.5%) across LSOAs (Table 2; Figure 1). The age and sex group with the lowest estimated prevalence of chronic pain was women aged 35–44 (22.6%) and the highest prevalence was found in women aged 85+ (54.5%) (Table S7). Across IMD deciles, the lowest estimated prevalence of chronic pain was found for the most affluent decile (IMD 10) (23.2%) and the highest prevalence was found in the least affluent decile (IMD 1) (46.5%) (Table S8). Prevalence of chronic pain was estimated as 24.6% among those living in rural LSOAs compared to 35.9% among those living in urban LSOAs (Table S5).

**TABLE 2:** Prevalence and variation in chronic pain and high‐impact chronic pain in neighbourhoods within local authorities in North Staffordshire.

	Local authority		
	Stoke‐on‐Trent	Newcastle‐under‐Lyme	Staffordshire moorlands
Population aged 35 years and over^a^	136,718	74,399	64,274
No. of MSOAs	34	16	13
No. of LSOAs	159	80	59
Chronic pain			
Overall *N*	52,699	23,103	19,171
Overall prevalence (%)	38.5	31.1	29.8
MSOA‐specific prevalence (%): median (range)	38.5 (28.8–48.1)	31.4 (23.0–39.5)	29.9 (26.3–36.6)
LSOA‐specific prevalence (%): median (range)	39.9 (24.5–50.1)	30.9 (18.6–47.3)	30.5 (21.7–42.7)
No. of LSOAs in lowest quintile of prevalence^b^	9	25	26
No. of LSOAs in highest quintile of prevalence	56	4	0
20:20^c^	0.30	0.26	0.44
High‐impact chronic pain			
Overall *N*	23,341	8723	6839
Overall prevalence (%)	17.1	11.7	10.6
MSOA‐specific prevalence (%): median (range)	16.7 (9.7–26.8)	11.3 (8.5–17.5)	10.7 (8.0–14.8)
LSOA‐specific prevalence (%): median (range)	17.1 (8.0–33.1)	11.3 (6.8–25.7)	10.2 (6.2–23.1)
No. of LSOAs in lowest quintile of prevalence	10	24	26
No. of LSOAs in highest quintile of prevalence	54	4	2
20:20	0.28	0.25	0.41

Abbreviations: LSOA, Lower Layer Super Output Area; MSOA, Middle Layer Super Output Area.

^a^ Mid‐2017 (Source: Office for National Statistics).

^b^ From LSOA‐specific estimates across North Staffordshire & Stoke‐on‐Trent.

^c^ Calculated as (no. of LSOAs in highest quintile − no. of LSOAs in lowest quintile)/total no. of LSOAs (atlas_of_inequality_18_nov_2019_FINAL.pdf(nuffieldfoundation.org)).

### 3.4 Estimated prevalence of high‐impact chronic pain

The estimated prevalence of high‐impact chronic pain across LSOAs ranged from 6.2% (95% confidence interval [1.7, 16.0]) to 33.1% [13.3, 44.7] (median 12.2%; Figure 1).

The prevalence of high‐impact chronic pain in IMD deciles ranged from 9.0% [5.9, 11.4] in the second most affluent decile (IMD 9) to 24.7% [19.0, 29.0] in the least affluent decile (IMD 1) (Tables S9 and S10). An exploratory secondary analysis, in which we replaced the age.sex interaction with age.IMD (quintile) confirmed that such inequalities appeared present across all age groups (Table S12). The prevalence of high‐impact chronic pain tended to be higher among people living in urban LSOAs than rural LSOAs (Table S6).

### 3.5 Sensitivity analyses

In the majority of LSOAs, the prevalence estimates were similar with and without the application of correction factors for ethnicity; corrected estimates of chronic pain prevalence ranged from −0.03% to 2.61% higher and −0.02% to 2.60% higher for high‐impact chronic pain among the 298 LSOAs (Figures S2–S5). For both chronic pain and high‐impact chronic pain, there were 15 LSOAs for which the estimated prevalence increased by 1% or more.

Compared to the main multilevel model for analysis of high‐impact chronic pain, the residual (unexplained) variance in the propensity to have high‐impact chronic pain that was attributable to unobserved LSOA characteristics was slightly lower with the inclusion of BMI in the multilevel model, with a more marked decrease upon inclusion of depression in the model (Table 3).

**TABLE 3:** Residual variance in multilevel models of high‐impact chronic pain, chronic pain, and sensitivity analysis models.

Main analysis (sample size = 4162)	
Outcome	Residual variance
High‐impact chronic pain	4.17%
Chronic pain	0.96%

Model sensitivity analyses (sample size = 3323)		
Outcome	Additional predictor(s)	Residual variance
High‐impact chronic pain	*[null model]*	6.49%
High‐impact chronic pain	*[main model]*	2.91%
High‐impact chronic pain	*[main model]* + BMI	2.13%
High‐impact chronic pain	*[main model]* + depression	1.02%
High‐impact chronic pain	*[main model]* + depression + BMI	1.09%

Abbreviation: BMI, body mass index.

## 4 DISCUSSION

### 4.1 Summary of main findings

Our study found substantial variation in the prevalence of chronic pain between neighbourhoods within local authorities. Using multilevel modelling and post‐stratification and an ethnicity correction factor applied to local survey data on over‐35‐year‐olds, we found two to threefold variation in the prevalence of chronic pain between the most extreme neighbourhood estimates within the same local authority. Inequalities were more pronounced for high‐impact chronic pain, with a fivefold variation between extremes.

### 4.2 Comparison with previous literature

Overall our prevalence estimate of 34.4% of adults aged 35 years and over experiencing chronic pain is low compared to previous population surveys of adults in the UK (35–51%; Fayaz et al., 2016), including estimates from the Health Survey for England carried out in the same year (PHE, 2017). This reflects our choice of the more stringent National Pain Survey definition of chronic pain which excludes ‘intermittent’ pain and requires that pain be present on most/all days. Selective non‐response among men with chronic pain aged 85 years and over may also have contributed although the magnitude of bias would be small given they constitute 1.4% of the target population. The higher prevalence of chronic pain with older age, female sex, and neighbourhood deprivation that underpinned our models is already well‐described. The higher prevalence of chronic and high‐impact chronic pain in urban areas is in contrast to a previous survey of adults aged over 55 years in Scotland which found no or modest differences in the opposite direction for regional and chronic widespread pain respectively (Docking et al., 2015).

There are no directly comparable estimates of chronic pain at LSOA level in the UK. However, when aggregated to the level of local authority, our estimates suggest greater differences in prevalence between the three local authorities in our study than currently available estimates of musculoskeletal painful disorders (Table S11).

### 4.3 Strengths and limitations

Using multilevel modelling with poststratification, we were able to estimate chronic and high‐impact chronic pain prevalence within North Staffordshire using local data with prohibitively small sample sizes per LSOA for direct estimation of health indicators at small area level. Whilst our analyses provide new insight into the distribution of the burden of chronic and high‐impact chronic pain in North Staffordshire, findings may not be generalizable to other areas. Following very low response rates among 25–34 years in our pilot study, we restricted our main survey to 35+ years for efficient use of resources and to reduce the potential for underpowered and biased estimates in this younger age category. Mechanisms producing the variation between LSOAs were not explored. National diversity in ethnicity is unlikely to be reflected in the data due to a high proportion of people identifying as White. Individual‐level ethnicity variables could not be modelled due to the lack of available tabulation of population‐level data by ethnicity as well as the other modelled variables for the poststratification stage. Variables reflecting LSOA‐level ethnic diversity could not be included in the multilevel models due to a combination of missing data on ethnicity and small numbers of respondents identifying as Black, Asian or Mixed/Multiple ethnicity. We applied correction factors to reduce bias affecting LSOA prevalence estimates based on prevalence estimates by ethnic group for pain‐related variables available from other publications and data sources (Allison et al., 2002; Macfarlane et al., 2015; Nicholl et al., 2015; PHE, 2017; University of Essex, 2022). For high‐impact chronic pain, we chose to use the least conservative values suggested by the available resources as correction factors given some evidence of greater disparities for more severe pain. Despite this, corrected and uncorrected estimates prevalence estimates were generally similar reflecting the high proportion of people from White backgrounds making up the North Staffordshire population.

It was not feasible to generate confidence intervals for LSOA prevalence estimates of chronic pain due to the computation time. Confidence intervals for high‐impact chronic pain estimates were generated using 5000 bootstrap replicates, with each replicate computing in approximately 1 h. Bootstrap attempts using fewer replicates were insufficient to generate confidence intervals.

### 4.4 Implications

Our study demonstrates a method for producing small‐area estimates of potential need that has hitherto been lacking. The approach may provide the flexibility for local health authorities to map local health variation using available local data (Zhang et al., 2014). This study indicates the need to use local‐level data to understand health needs and guide interventions to improve population health and reduce inequalities. Scalable options in the future may be able to exploit primary care EHR data as approaches to recording and classifying chronic pain develop. Further work is required to identify specific reasons for inequalities at the local level. Harnessing routinely collected data is the first stage in exploring whether the complex array of factors that lead to inequalities can be identified, although often survey data is required to provide information on health function and lifestyle.

Despite encouragement from the Chief Medical Officer for England (Chief Medical Officer, 2008), neither chronic pain nor any of the common musculoskeletal conditions associated with it were ever included in the Quality and Outcomes Framework (QOF). Geographical variation in opioid analgesic prescription is well‐documented (Schifanella et al., 2020), but whilst this indicator is associated with pain at population and individual levels (Asaria et al., 2016), there is sufficient discordance between the two to question its suitability as a reliable proxy for the prevalence of chronic pain within defined populations. Such indicators tend also to frame the issue within a narrower healthcare service perspective rather than one based on the experience of chronic pain and in which it is easier to recognize the role of determinants beyond healthcare.

Small‐area variation in chronic pain has not previously been systematically described. With an ageing population and increasing inequalities, the incidence and prevalence of chronic and high‐impact chronic pain will make an increasing contribution to reduced population health and increasing demand on health services. Substantial inequalities between neighbourhoods imply that some levelling‐up actions by integrated care systems/boards could be targeted at communities with the greatest need living in the most deprived neighbourhoods and the services that currently serve them, including general practices and community pharmacies. The lower estimates may indicate the level that can be achieved. Similarly, with regard to working‐age adults (see Table S12), the prevalence of high‐impact chronic pain is notably higher in the most deprived quintile. This suggests there is a need for strategies to reduce pain impact particularly in areas of high deprivation and to provide employment opportunities and an environment to maintain work participation despite the presence of pain (Wilkie et al., 2013).

Approaches to develop local economies, regenerate deprived areas and implementation of effective health improvement programmes with improved healthcare are important (Steel et al., 2018). In the absence of rich, local data, the existing CORE20PLUS approach (a UK‐wide approach targeting the 20% most deprived areas in the population) may be a reasonable proxy for targeting communities with the greatest need; whilst pain is not one of the five areas of focus, this approach will target the communities with higher levels of pain (NHS England, n.d.). Relevant to pain, the NHS Long Term Plan highlights the roll‐out of First Contact Practitioners, expanding the physiotherapist workforce in primary care, and expanding access to effective interventions such as ESCAPE‐pain, including new digital versions. However, the extent to which these initiatives are being targeted to individuals and communities with the highest levels of need is unclear. Workforce shortages in primary care disproportionately affect deprived areas and the ability to implement new strategies is more challenging (Nussbaum et al., 2021). Policy initiatives to ensure there is capacity to deliver healthcare initiatives are important alongside approaches to build social capital and a stronger public health approach to prevention and health promotion will be key to reducing the frequency of chronic and high‐impact pain (The Academy of Medical Sciences, 2016).

## AUTHOR CONTRIBUTIONS

George Peat and Ross Wilkie conceived the research study. Marty Lynch conducted the analysis and produced results tables and figures with input from George Peat, Dahai Yu, Kelvin Jordan, and Ross Wilkie. All authors contributed to analysis decisions, interpretation of results, and writing of the manuscript.

## FUNDING INFORMATION

This study was funded by project funding from Versus Arthritis for the PRELIM Survey (21403) and from Nuffield Foundation and Versus Arthritis for the MIDAS Project (OBF/43390). Visit http://www.nuffieldfoundation.org/. The views expressed are those of the authors and not necessarily the funders. KJ, RW and DY hold Honorary Academic Consultant Contracts from Office for Health Improvement and Disparities. KJ is supported by matched funding awarded to the NIHR Applied Research Collaboration (West Midlands).

## CONFLICT OF INTEREST STATEMENT

The authors declare that there are no conflicts of interest.

## Supporting information


Appendix S1.
