# Supplementary material for: Where does it hurt? Small area estimates and inequality in the prevalence of chronic pain
Source: Eur J Pain. 2023 Jun 21;27(10):1177–86. doi: 10.1002/ejp.2148 (PMC10947147; doi:10.1002/ejp.2148)

**Where does it hurt? Small area estimates and inequality in the prevalence of chronic pain**

Marty Lynch, George Peat, Kelvin Jordan, Dahai Yu, Ross Wilkie

**SUPPLEMENTARY MATERIAL**

Table S1: Patterns of missingness in variables used for analysis. By definition, all respondents with complete high impact chronic pain data also had complete chronic pain data.

Table S2: Patterns of missingness in variables used for analysis and sensitivity analyses. By definition, all respondents with complete high impact chronic pain data also had complete chronic pain data.

Table S3: Number or respondents (n=4162) by sex, age group, and IMD decile (where IMD 1 is least affluent and IMD 10 is most affluent)

Table S4: Estimated coefficients for multilevel models of high impact chronic pain and chronic pain

Table S5: Estimated prevalence of chronic pain by rurality of LSOA in North Staffordshire

Table S6: Estimated prevalence of high impact chronic pain by rurality of LSOA in North Staffordshire with 95% confidence intervals

Table S7: Estimated prevalence of high impact chronic pain by age and sex in North Staffordshire with 95% confidence intervals

Table S8: Estimated prevalence of high impact chronic pain by IMD in North Staffordshire with 95% confidence intervals

Table S9: Estimated prevalence of chronic pain by age and sex in North Staffordshire

Table S10: Estimated prevalence of chronic pain by IMD decile of LSOA in North Staffordshire

Table S11: Comparison of pain prevalence estimates at the level of local authority from the current survey and from existing national data sources

Table S12: Estimated coefficients for secondary analysis multilevel model of high impact chronic pain including age and IMD quintile interaction

Figure S1: Map showing the number of respondents within each LSOA in North Staffordshire

Figure S2: Scatter plot of high impact chronic (HICP) pain prevalence estimates without correction for ethnicity (x-axis) and with correction for ethnicity (y-axis) for each LSOA

Figure S3: Scatter plot of chronic pain prevalence estimates without correction for ethnicity (x-axis) and with correction for ethnicity (y-axis) for each LSOA

Figure S4: Map of the estimated prevalence of chronic pain in LSOAs in North Staffordshire without correction for ethnicity

Figure S5: Map of the estimated prevalence of high impact chronic pain in LSOAs in North Staffordshire without correction for ethnicity

Separate file: ‘LSOA results supplementary material table.csv’ Results for chronic and high impact chronic pain (with ethnicity correction) for each LSOA in North Staffordshire

Table S1: Patterns of missingness in variables used for analysis. By definition, all respondents with complete high impact chronic pain data also had complete chronic pain data.

| Rural | Age group | Sex | High impact chronic pain | Number of respondents |
| --- | --- | --- | --- | --- |
| complete | complete | complete | complete | 4162 |
| complete | complete | complete | **missing** | 142 |
| complete | complete | **missing** | complete | 46 |
| complete | complete | **missing** | **missing** | 4 |
| complete | **missing** | complete | complete | 25 |
| complete | **missing** | complete | **missing** | 1 |
| complete | **missing** | **missing** | complete | 8 |
| complete | **missing** | **missing** | **missing** | 1 |

Table S2: Patterns of missingness in variables used for analysis and sensitivity analyses. By definition, all respondents with complete high impact chronic pain data also had complete chronic pain data.

| Rural | Age group | Sex | High impact chronic pain | Depression | BMI | Number of respondents |
| --- | --- | --- | --- | --- | --- | --- |
| complete | complete | complete | complete | complete | complete | 3323 |
| complete | complete | complete | complete | complete | **missing** | 308 |
| complete | complete | complete | complete | **missing** | complete | 2 |
| complete | complete | complete | complete | **missing** | **missing** | 529 |
| complete | complete | complete | **missing** | complete | complete | 98 |
| complete | complete | complete | **missing** | complete | **missing** | 21 |
| complete | complete | complete | **missing** | **missing** | **missing** | 23 |
| complete | complete | **missing** | complete | complete | complete | 35 |
| complete | complete | **missing** | complete | complete | **missing** | 11 |
| complete | complete | **missing** | **missing** | complete | complete | 3 |
| complete | complete | **missing** | **missing** | complete | **missing** | 1 |
| complete | **missing** | complete | complete | complete | complete | 19 |
| complete | **missing** | complete | complete | **missing** | **missing** | 6 |
| complete | **missing** | complete | **missing** | complete | complete | 1 |
| complete | **missing** | **missing** | complete | complete | complete | 6 |
| complete | **missing** | **missing** | complete | complete | **missing** | 1 |
| complete | **missing** | **missing** | complete | **missing** | **missing** | 1 |
| complete | **missing** | **missing** | **missing** | **missing** | **missing** | 1 |

Table S3: Number or respondents (n=4162) by sex, age group, and IMD decile (where IMD 1 is least affluent and IMD 10 is most affluent).

|  |  | IMD 1 | IMD 2 | IMD 3 | IMD 4 | IMD 5 | IMD 6 | IMD 7 | IMD 8 | IMD 9 | IMD 10 |
| --- | --- | --- | --- | --- | --- | --- | --- | --- | --- | --- | --- |
| Women |  |  |  |  |  |  |  |  |  |  |  |
|  | 35-44 | 16 | 19 | 20 | 17 | 18 | 19 | 34 | 27 | 21 | 7 |
|  | 45-54 | 40 | 33 | 32 | 33 | 34 | 61 | 72 | 74 | 54 | 18 |
|  | 55-64 | 47 | 36 | 29 | 42 | 64 | 67 | 82 | 77 | 86 | 28 |
|  | 65-74 | 48 | 52 | 43 | 55 | 68 | 67 | 132 | 102 | 90 | 36 |
|  | 75-84 | 29 | 30 | 18 | 24 | 29 | 46 | 63 | 63 | 51 | 15 |
|  | 85+ | 16 | 8 | 6 | 5 | 9 | 7 | 9 | 13 | 12 | 5 |
| Men |  |  |  |  |  |  |  |  |  |  |  |
|  | 35-44 | 7 | 6 | 15 | 12 | 11 | 13 | 17 | 8 | 5 | 9 |
|  | 45-54 | 26 | 22 | 24 | 18 | 29 | 51 | 45 | 33 | 47 | 18 |
|  | 55-64 | 30 | 34 | 36 | 32 | 48 | 54 | 71 | 66 | 47 | 19 |
|  | 65-74 | 36 | 38 | 36 | 36 | 42 | 78 | 98 | 83 | 77 | 31 |
|  | 75-84 | 16 | 20 | 14 | 32 | 31 | 38 | 46 | 61 | 45 | 22 |
|  | 85+ | 9 | 4 | 5 | 9 | 5 | 4 | 14 | 8 | 10 | 3 |

Note: 510 (12%) of 4162 respondents lived in rural areas and 3651 respondents (88%) lived in urban areas

Table S4: Estimated coefficients for multilevel models of high impact chronic pain and chronic pain

|  | *Chronic pain* | | | |  | *High impact chronic pain* | | | |
| --- | --- | --- | --- | --- | --- | --- | --- | --- | --- |
|  | Coefficient | Standard error | p-value | |  | Coefficient | Standard error | p-value | |
| Intercept | -1.67 | 0.3 | <0.01 |  |  | -3.24 | 0.54 | <0.01 |  |
| AgeSex:35to44F | -0.14 | 0.3 | 0.65 |  |  | 0.28 | 0.54 | 0.61 |  |
| AgeSex:45to54F | 0.52 | 0.27 | 0.05 |  |  | 0.84 | 0.48 | 0.08 |  |
| AgeSex:55to64F | 0.65 | 0.26 | 0.01 |  |  | 1.18 | 0.47 | 0.01 |  |
| AgeSex:65to74F | 0.78 | 0.26 | <0.01 |  |  | 1.25 | 0.47 | <0.01 |  |
| AgeSex:75to84F | 1.09 | 0.27 | <0.01 |  |  | 1.88 | 0.47 | <0.01 |  |
| AgeSex:85plusF | 1.37 | 0.33 | <0.01 |  |  | 1.92 | 0.52 | <0.01 |  |
| AgeSex:45to54M | 0.31 | 0.28 | 0.27 |  |  | 0.49 | 0.5 | 0.33 |  |
| AgeSex:55to64M | 0.48 | 0.27 | 0.07 |  |  | 0.78 | 0.48 | 0.1 |  |
| AgeSex:65to74M | 0.71 | 0.26 | <0.01 |  |  | 1.32 | 0.47 | <0.01 |  |
| AgeSex:75to84M | 0.97 | 0.27 | <0.01 |  |  | 1.73 | 0.48 | <0.01 |  |
| AgeSex:85plusM | 0.4 | 0.36 | 0.27 |  |  | 1.34 | 0.56 | 0.02 |  |
| IMD1 | 1.06 | 0.21 | <0.01 |  |  | 1.19 | 0.31 | <0.01 |  |
| IMD2 | 0.88 | 0.22 | <0.01 |  |  | 0.91 | 0.32 | <0.01 |  |
| IMD3 | 0.6 | 0.22 | <0.01 |  |  | 0.21 | 0.34 | 0.53 |  |
| IMD4 | 0.35 | 0.22 | 0.11 |  |  | 0.23 | 0.34 | 0.49 |  |
| IMD5 | 0.37 | 0.21 | 0.09 |  |  | 0.18 | 0.33 | 0.59 |  |
| IMD6 | 0.49 | 0.21 | 0.02 |  |  | 0.19 | 0.32 | 0.55 |  |
| IMD7 | 0.35 | 0.2 | 0.08 |  |  | 0.15 | 0.31 | 0.63 |  |
| IMD8 | 0.29 | 0.2 | 0.15 |  |  | 0.05 | 0.32 | 0.87 |  |
| IMD9 | 0.2 | 0.2 | 0.32 |  |  | -0.11 | 0.32 | 0.73 |  |
| Rural | -0.35 | 0.13 | <0.01 |  |  | -0.31 | 0.21 | 0.14 |  |

Table S5: Estimated prevalence of chronic pain by rurality of LSOA in North Staffordshire

|  |  | Chronic pain prevalence, % | |
| --- | --- | --- | --- |
| Rurality |  | *without ethnicity adjustment* | *with ethnicity adjustment* |
| Urban |  | 35.76 | 35.93 |
| Rurality |  | 24.56 | 24.58 |

Table S6: Estimated prevalence of high impact chronic pain by rurality of LSOA in North Staffordshire with 95% confidence intervals

|  | High impact chronic pain prevalence (95% CI), % | |
| --- | --- | --- |
| Rurality | *without ethnicity adjustment* | *with ethnicity adjustment* |
| Urban | 14.76 (12.58,15.48) | 14.93 (12.72,15.66) |
| Rurality | 8.60 (5.54,11.29) | 8.61 (5.55,11.31) |

Table S7: Estimated prevalence of high impact chronic pain by age and sex in North Staffordshire with 95% confidence intervals

|  |  | High impact chronic pain prevalence (95% CI), % | |
| --- | --- | --- | --- |
| Women | | *without ethnicity adjustment* | *with ethnicity adjustment* |
|  | 35-44 | 8.05 (3.75,12.02) | 8.17 (3.81,12.2) |
|  | 45-54 | 12.37 (8.93,15.22) | 12.51 (9.03,15.40) |
|  | 55-64 | 16.01 (12.02,18.05) | 16.17 (12.13,18.24) |
|  | 65-74 | 16.37 (12.72,18.42) | 16.51 (12.83,18.58) |
|  | 75-84 | 26.64 (21.42,30.78) | 26.87 (21.62,31.04) |
|  | 85+ | 27.86 (18.39,37.77) | 28.11 (18.57,38.09) |
| Men | |  |  |
|  | 35-44 | 6.33 (1.41,10.68) | 6.43 (1.44,10.85) |
|  | 45-54 | 9.25 (5.39,11.95) | 9.37 (5.46,12.10) |
|  | 55-64 | 11.61 (7.91,13.94) | 11.74 (8.00,14.09) |
|  | 65-74 | 17.41 (13.34,19.93) | 17.57 (13.46,20.12) |
|  | 75-84 | 23.55 (17.83,27.79) | 23.76 (18.00,28.02) |
|  | 85+ | 17.61 (8.39,26.06) | 17.78 (8.47,26.29) |

Table S8: Estimated prevalence of high impact chronic pain by IMD in North Staffordshire with 95% confidence intervals

|  | High impact chronic pain prevalence (95% CI), % | |
| --- | --- | --- |
| IMD decile | *without ethnicity adjustment* | *with ethnicity adjustment* |
| 1 (Least affluent) | 24.20 (18.58,28.45) | 24.71 (18.97,29.02) |
| 2 | 19.75 (14.79,24.78) | 20.04 (15.02,25.13) |
| 3 | 11.88 (7.27,14.96) | 11.97 (7.33,15.08) |
| 4 | 11.84 (7.50,15.02) | 11.92 (7.55,15.11) |
| 5 | 11.60 (6.83,13.38) | 11.64 (6.86,13.43) |
| 6 | 11.28 (7.55,13.92) | 11.32 (7.57,13.96) |
| 7 | 11.06 (8.37,14.67) | 11.10 (8.39,14.72) |
| 8 | 9.95 (6.49,11.74) | 9.98 (6.50,11.76) |
| 9 | 8.92 (5.87,11.33) | 8.96 (5.90,11.38) |
| 10 (Most affluent) | 9.76 (4.86,13.62) | 9.80 (4.87,13.67) |

Table S9: Estimated prevalence of chronic pain by age and sex in North Staffordshire

|  |  | Chronic pain prevalence, % | |
| --- | --- | --- | --- |
| Women | | *without ethnicity adjustment* | *with ethnicity adjustment* |
|  | 35-44 | 22.50 | 22.63 |
|  | 45-54 | 34.42 | 34.57 |
|  | 55-64 | 36.92 | 37.07 |
|  | 65-74 | 39.15 | 39.29 |
|  | 75-84 | 47.10 | 47.27 |
|  | 85+ | 54.33 | 54.52 |
| Men | |  |  |
|  | 35-44 | 25.17 | 25.34 |
|  | 45-54 | 30.16 | 30.32 |
|  | 55-64 | 33.33 | 33.47 |
|  | 65-74 | 37.66 | 37.80 |
|  | 75-84 | 43.50 | 43.66 |
|  | 85+ | 30.82 | 30.95 |

Table S10: Estimated prevalence of chronic pain by IMD decile of LSOA in North Staffordshire

|  | Chronic pain prevalence, % | |
| --- | --- | --- |
| IMD decile | *without ethnicity adjustment* | *with ethnicity adjustment* |
| 1 (Least affluent) | 46.03 | 46.51 |
| 2 | 41.94 | 42.22 |
| 3 | 36.46 | 36.59 |
| 4 | 30.50 | 30.59 |
| 5 | 30.96 | 31.02 |
| 6 | 32.60 | 32.65 |
| 7 | 30.43 | 30.48 |
| 8 | 28.38 | 28.41 |
| 9 | 27.99 | 28.05 |
| 10 (Most affluent) | 23.17 | 23.20 |

Table S11: Comparison of pain prevalence estimates at the level of local authority from the current survey and from existing national data sources

| **Data source and year** | **Scope** | **Indicator** | **Target population** | **Stoke-on-Trent** | **Newcastle-under-Lyme** | **Staffordshire Moorlands** |
| --- | --- | --- | --- | --- | --- | --- |
| PRELIM Survey, 2017 | Local | Chronic pain | Over-35s | 38.5 | 31.1 | 29.8 |
| PRELIM Survey, 2017 | Local | High impact chronic pain | Over-35s | 17.1 | 11.7 | 10.6 |
|  |  |  |  |  |  |  |
| GPPS, 2020 | National | Long-term musculoskeletal problem | Over-16s | 22.0 | 25.2 | 24.4 |
| MSK Calculator/HSfE, 2012 | National | Back pain | All ages | 17.6 | 17.6 | 18.8 |
| MSK Calculator/HSfE, 2012 | National | Severe back pain | All ages | 11.3 | 11.2 | 11.8 |
| MSK Calculator/HSfE, 2012 | National | Hip osteoarthritis | Over-45s | 11.5 | 10.7 | 11.2 |
| MSK Calculator/HSfE, 2012 | National | Severe hip osteoarthritis | Over-45s | 3.6 | 3.3 | 3.3 |
| MSK Calculator/HSfE, 2012 | National | Knee osteoarthritis | Over-45s | 19.7 | 17.9 | 18.8 |
| MSK Calculator/HSfE, 2012 | National | Severe knee osteoarthritis | Over-45s | 7.3 | 6.4 | 6.3 |
| **GPPS** GP Patient Survey; **HSfE** Health Survey for England; **MSK** Musculoskeletal | | | | | | |

Table S12: Estimated coefficients for secondary analysis multilevel model of high impact chronic pain including age and IMD quintile interaction

|  | Coefficient | Standard error | p-value | |
| --- | --- | --- | --- | --- |
| Intercept | -58.08 | 1.09 | <0.01 |  |
| Male | -0.17 | 0.09 | 0.07 |  |
| QIMD1.Age35to44 | 1.51 | 0.45 | <0.01 |  |
| QIMD1.Age45to54 | 2.30 | 0.22 | <0.01 |  |
| QIMD1.Age55to64 | 2.83 | 0.20 | <0.01 |  |
| QIMD1.Age65to74 | 2.72 | 0.21 | <0.01 |  |
| QIMD1.Age75to84 | 3.21 | 0.25 | <0.01 |  |
| QIMD1.Age85plus | 2.75 | 0.40 | <0.01 |  |
| QIMD2.Age35to44 | 0.89 | 0.52 | 0.09 |  |
| QIMD2.Age45to54 | 1.01 | 0.38 | <0.01 |  |
| QIMD2.Age55to64 | 1.54 | 0.23 | <0.01 |  |
| QIMD2.Age65to74 | 2.08 | 0.22 | <0.01 |  |
| QIMD2.Age75to84 | 2.50 | 0.25 | <0.01 |  |
| QIMD2.Age85plus | 1.93 | 0.46 | <0.01 |  |
| QIMD3.Age35to44 | 0.39 | 0.51 | 0.45 |  |
| QIMD3.Age45to54 | 1.42 | 0.34 | <0.01 |  |
| QIMD3.Age55to64 | 1.48 | 0.27 | <0.01 |  |
| QIMD3.Age65to74 | 2.00 | 0.21 | <0.01 |  |
| QIMD3.Age75to84 | 2.22 | 0.23 | <0.01 |  |
| QIMD3.Age85plus | 3.35 | 0.54 | <0.01 |  |
| QIMD4.Age35to44 | 0.87 | 0.38 | <0.05 |  |
| QIMD4.Age45to54 | 1.49 | 0.23 | <0.01 |  |
| QIMD4.Age55to64 | 1.43 | 0.20 | <0.01 |  |
| QIMD4.Age65to74 | 1.77 | 0.18 | <0.01 |  |
| QIMD4.Age75to84 | 2.45 | 0.15 | <0.01 |  |
| QIMD4.Age85plus | 2.24 | 0.35 | <0.01 |  |
| QIMD5.Age45to54 | 0.41 | 0.38 | 0.28 |  |
| QIMD5.Age55to64 | 1.59 | 0.31 | <0.01 |  |
| QIMD5.Age65to74 | 1.65 | 0.21 | <0.01 |  |
| QIMD5.Age75to84 | 2.23 | 0.25 | <0.01 |  |
| QIMD5.Age85plus | 2.20 | 0.48 | <0.01 |  |
| Rural | -0.33 | 0.20 | 0.1 |  |

QIMD1 indicates most deprived IMD quintile and QIMD5 indicates least deprived quintile.

Figure S1: Map showing the number of respondents within each LSOA in North Staffordshire


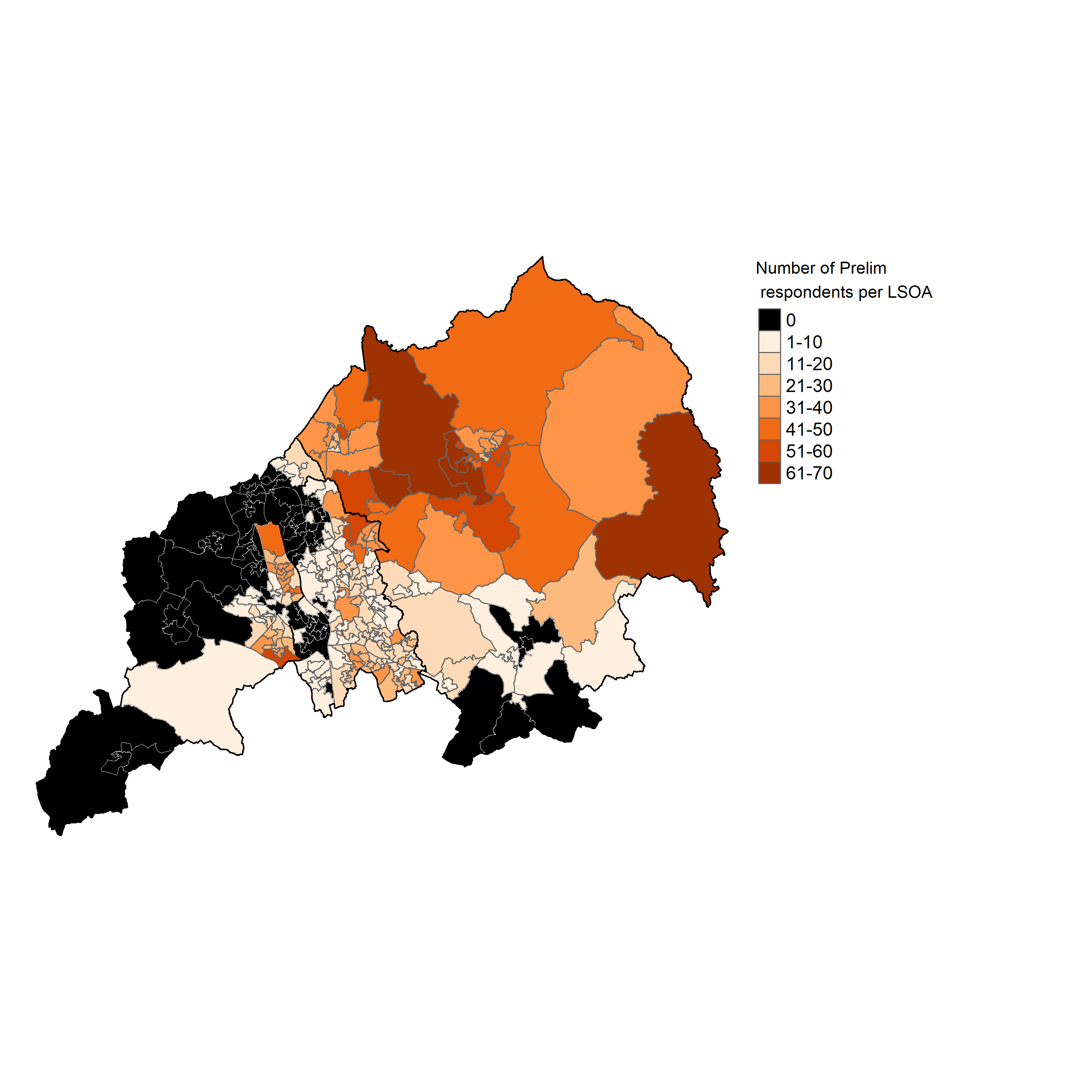


Figure S2: Scatter plot of high impact chronic (HICP) pain prevalence estimates without correction for ethnicity (x-axis) and with correction for ethnicity (y-axis) for each LSOA


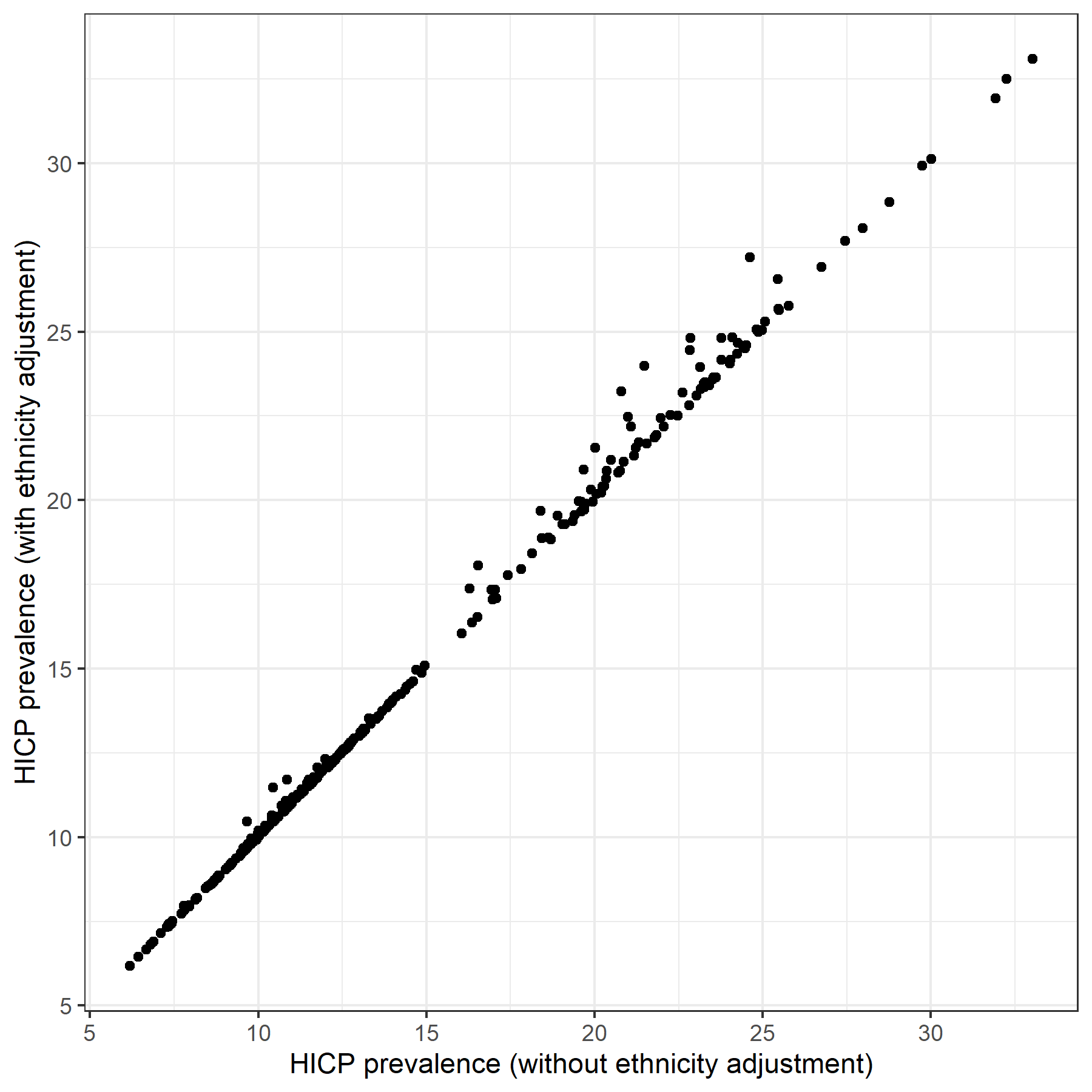


Figure S3: Scatter plot of chronic pain prevalence estimates without correction for ethnicity (x-axis) and with correction for ethnicity (y-axis) for each LSOA


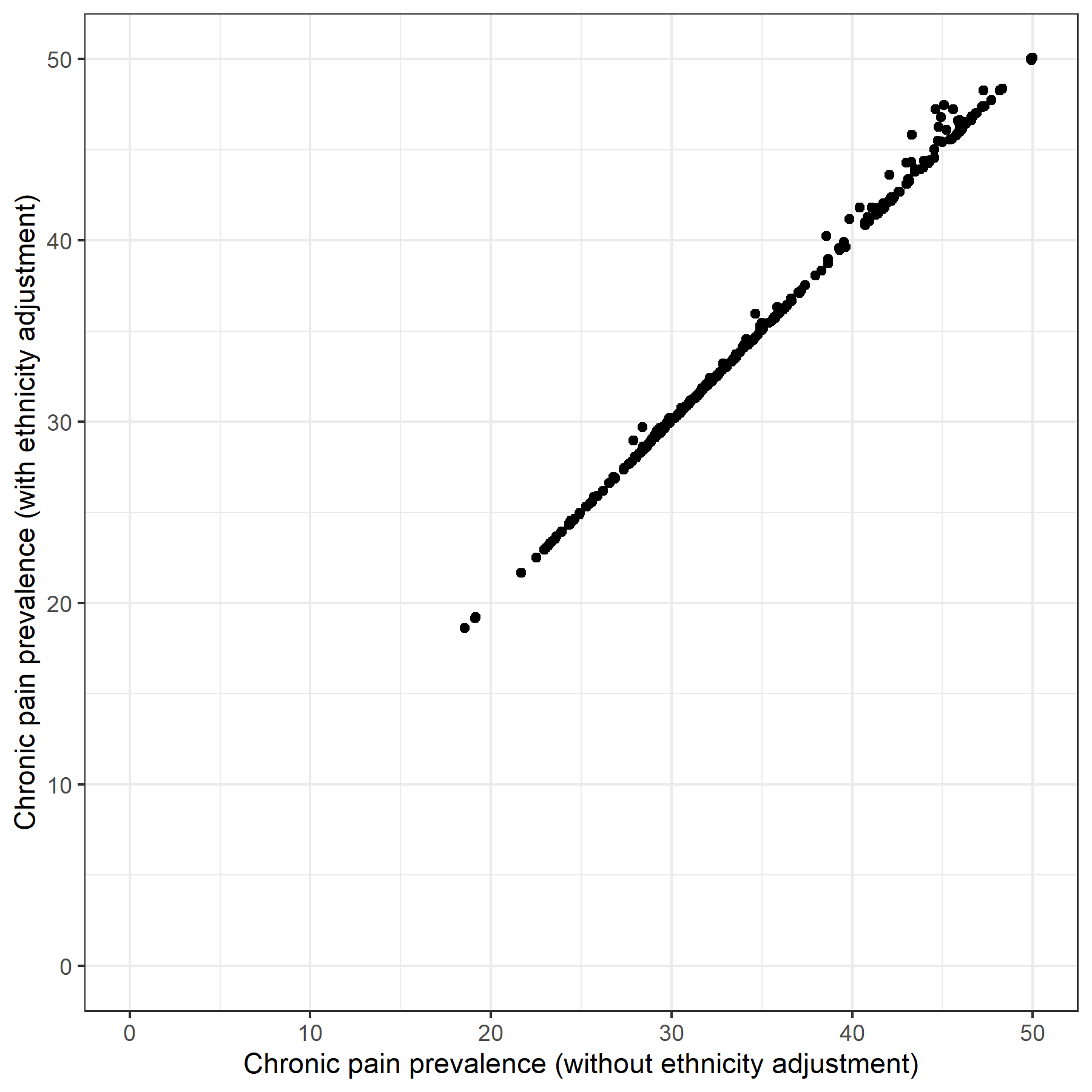


Figure S4: Map of the estimated prevalence of chronic pain in LSOAs in North Staffordshire without correction for ethnicity


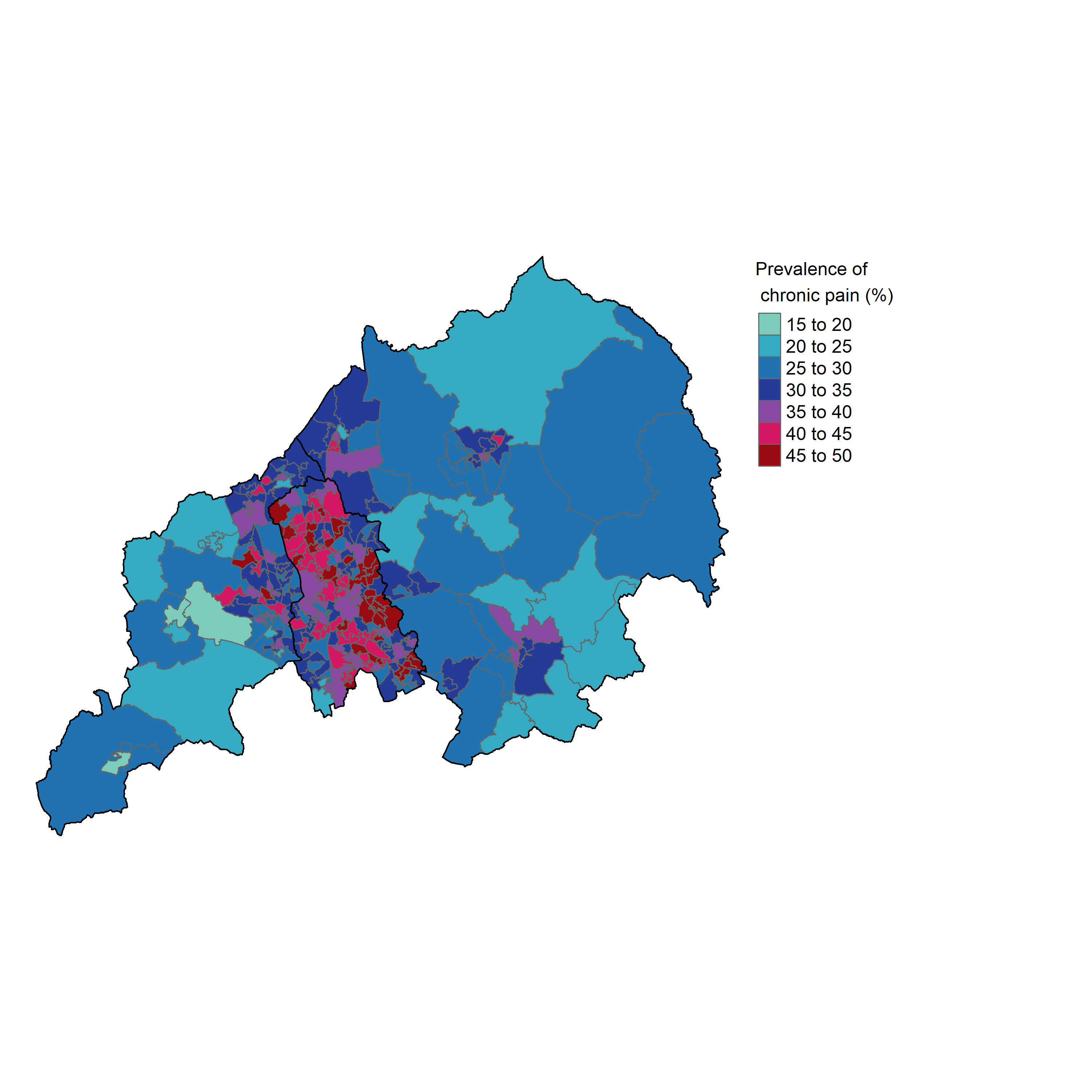


Figure S5: Map of the estimated prevalence of high impact chronic pain in LSOAs in North Staffordshire without correction for ethnicity


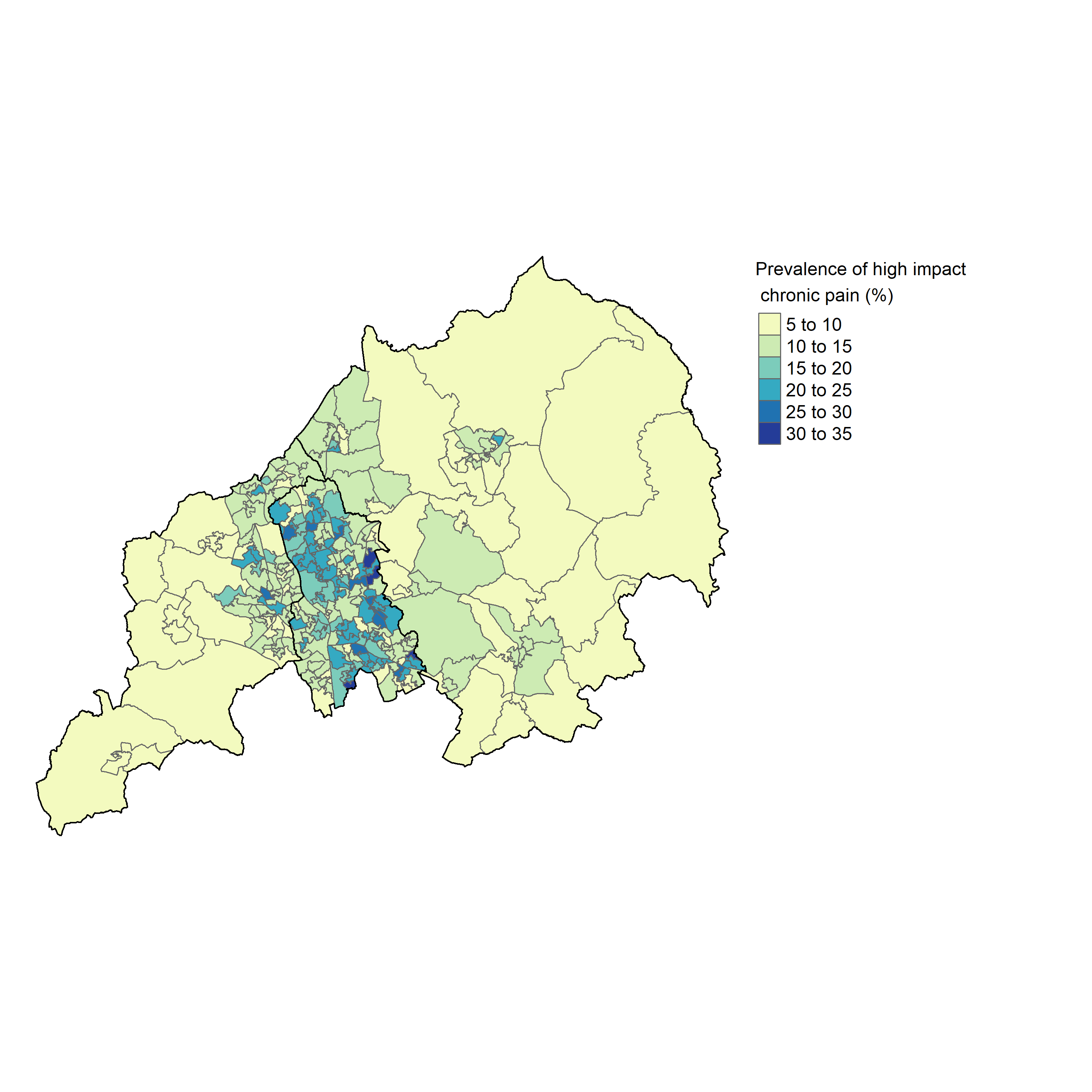

Supplement: Supplementary file 1 — Appendix S1. [file EJP-27-1177-s001.zip › Where does it hurt supplementary material - tracked changes.docx]
